# Lifestyle counselling as secondary prevention in patients with minor stroke and transient ischemic attack: study protocol for a randomized controlled pilot study

**DOI:** 10.1186/s40814-020-00583-4

**Published:** 2020-03-25

**Authors:** Jacob Liljehult, Stig Molsted, Tom Møller, Dorthe Overgaard, Lis Adamsen, Mary Jarden, Thomas Christensen

**Affiliations:** 1 Department of Neurology, Nordsjællands Hospital, Dyrehavevej 29, DK-3400 Hillerød, Denmark; 2 Department 9701, The University Hospitals Centre for Health Research UCSF, Copenhagen University Hospital (Rigshospitalet), Blegdamsvej 9, DK-2100 Copenhagen, Denmark; 3 Faculty of Health and Technology, Institute of Nursing and Nutrition, Copenhagen University College, Tagensvej 86, DK-2200 Copenhagen N, Denmark; 4 Department of Clinical Research, Nordsjællands Hospital, Dyrehavevej 29, DK-3400 Hillerød, Denmark; 5 Institute of Public Health, University of Copenhagen, Øster Farimagsgade 5, DK-1353 Copenhagen K, Denmark; 6 Department of Clinical Medicine, University of Copenhagen, Blegdamsvej 3, DK-2200 Copenhagen N, Denmark

**Keywords:** Stroke, Transient ischemic attack, Smoking, Exercise, Physical activity, Adherence, Early rehabilitation, Health counselling

## Abstract

**Background:**

Most patients with minor stroke or transient ischemic attack (TIA) are discharged with little or no specialised follow-up. Nonetheless, these patients have a high prevalence of cognitive impairments and a considerable risk of recurrent stroke. Smoking cessation, physical activity, and adherence to antihypertensive and antithrombotic medication are highly recommended in patients with minor stroke and TIA. Evidence suggests that simple encouragement to change lifestyle is ineffective. Behavioural interventions might therefore be needed to support patients in managing their own health post-discharge.

**Objectives:**

We aim to test the (1) feasibility of randomisation acceptance and an early initiated, client-centred lifestyle and behavioural intervention in a clinical setting, and (2) potential effect of the intervention on arterial blood pressure in patients with minor stroke or TIA and (3) explore the participants experience of barriers and facilitators for health behaviour after a stroke, including perceived needs and social support.

**Methods:**

We will conduct a randomized controlled pilot trial: Eligible patients with acute minor stroke or TIA (*n* = 40) will be randomly allocated to either early initiated counselling with four weekly post-discharge follow-up sessions for 12 weeks or usual care. The primary outcome will be program feasibility and to discuss the relevance of arterial blood pressure as primary outcome after 12 weeks intervention. Selected participants will be invited to participate in semi-structured interviews, based on purposeful sampling, to evaluate the intervention and explore their experience of life after a stroke. The interviews will be analysed using a five-step thematic analysis approach.

**Discussion:**

The study will provide evidence of the feasibility and potential effect of early initiated counselling on cardiovascular risk factors in patients with minor stroke and TIA. Qualitative interviews will contribute with a more nuanced understanding of the barriers and facilitators of health enhancing behaviour. Optimizing health behaviour counselling and providing formal support to the patients’ post-discharge may ease the transition and help more patients adhere to lifestyle and medication recommendations.

**Trial registration:**

ClinicalTrial.gov, NCT03648957

## Background

Stroke is a significant cause of morbidity, mortality, and disability in both western countries and globally [1]. In Denmark, the incidence rate is 3/1000 per year, with a mortality rate of 10% in the first month [2]. More than half of patients admitted with a stroke or transient ischemic attack (TIA) only have mild neurological symptoms and are often discharged after 3-5 days of hospital admission. In spite of this, there is evidence that the patients after discharge often experience cognitive and communicative impairments such as difficulties with everyday activities, memory, fatigue, reading and participating in conversations [3]. This indicates that patients with recent minor stroke or TIA need more support than previously assumed.

One in four patients admitted with acute stroke has previously suffered a stroke or TIA. The risk is greatest shortly after the incident stroke; and within the first year the recurrence rate is 12-13% and subsequently plateauing at 5-6% per year [4]. Recurrent stroke is an independent risk factor for loss of function, institutionalization and death [5].

In the past decades, there has been an increasing focus on health behaviour in relation to the prevention of vascular diseases [6]. Arterial hypertension is the most important and prevalent risk factor for stroke and TIA (odds ratio (OR) 2.64 [2.26-3.08]; Population Attributable Risk (PAR) 34.6%). However, other lifestyle factors, such as smoking (OR 2.09 [1.75-2.51]; PAR 18.9%), physical inactivity (OR 1.45 [1.11-1.89]; PAR 28.5%), abdominal obesity (OR 1.42 [1.18-1.71]; PAR 26.5%), and unhealthy eating habits (OR 1.35 [1.12-1.61]; PAR 18.8%) contribute to the risk of stroke [7].

Preventive medication is of utmost importance for secondary and tertiary prevention in patients with stroke and TIA. The majority of patients are treated with antithrombotic drugs or drugs to reduce blood pressure or blood lipids [8]. However, several studies have found that a substantial part of the patients discontinue the drug treatment overtime [9, 10].

The harmful effects derived from lifestyle factors, and the importance of adherence to preventive medication in relation to stroke is well-documented [10, 11]. But there is still insufficient knowledge regarding how to most effectively communicate this to the patients and how to support the patient in making suitable choices to prevent recurrent strokes and progression of the disease. The results from previous studies have been contradictory. It is difficult to identify the most effective components and recommend specific interventions or components of interventions for implementation in clinical practice. Lawrence et al. [12] found in a systematic review of 20 randomized controlled trials that multimodal behavioural interventions had a beneficial effect on blood pressure in patients with stroke. Deijle et al. [13] found that lifestyle interventions had a greater effect if the intervention contained elements of physical activity. Lager et al. [14] on the contrary found no significant reduction in cardiovascular events in a systematic review of 26 randomized controlled trials of lifestyle intervention for stroke patients.

The time of inclusion in previous stroke/lifestyle intervention studies varied from a few days after stroke onset [15, 16] to several years [17]. The timing of the intervention might affect the patient’s motivation and ability to adhere to the intervention, which will ultimately have an effect on the outcome. In other patient groups, it has been hypothesised that the time just after the diagnosis constitutes a certain *window of opportunity*; i.e. a limited period of time in which the patient is particularly receptive to information and behavioural changes [18].

The hypothesis of the present study is that early client-centred patient counselling with repeated follow-up sessions after discharge can reduce the blood pressure through smoking cessation, physical activity, and improved adherence to preventive medication in patients with minor stroke or TIA compared to simple encouragement to lifestyle change. The overall purpose of our research is to develop effective and clinically feasible interventions to support patients with minor stroke and TIA in engaging in health enhancing behaviour and ultimately prevent recurrent strokes.

Little research has explored how old age and cognitive impairments of this patient group affects their ability, readiness and willingness to participate in early initiated health behavioural interventions or if there are particular circumstances both during admission and post-discharge that should be taken into account when designing this type of research study.

The aims of this study are the following:

1. to evaluate the feasibility of a client-centred patient counselling intervention focused on smoking cessation, physical activity, and adherence to preventive medication in patients with minor stroke or TIA
2. to test the potential effects of the intervention on blood pressure and other cardiovascular risk factors in patients with minor stroke or TIA, and estimate means and standard deviations for subsequent sample-size calculations
3. to explore and evaluate the perceived needs and experience of social support of the participants in relation to health behavioural changes

## Methods

The study will consist of a parallel group randomized controlled feasibility trial and a qualitative interview study. Participants will be randomly allocated to either an individual face-to-face counselling intervention, with follow-up sessions post-discharge at 4 week intervals for 12 weeks, or usual care. Semi-structured qualitative interviews will be conducted with selected participants after the last follow-up.

### Setting and participants

The target population is hospitalised patients with recent minor stroke or TIA who are discharged home from the hospital.

We will include patients with acute minor stroke or TIA admitted to the Department of Neurology at Nordsjællands Hospital (*n* = 40). Nordsjællands Hospital is a university hospital with a catchment area of 310,000 citizens. All patients with stroke or TIA in the catchment area are admitted to the hospital and treatment is free of charge. The Department of Neurology has a specialized unit for patients with acute stroke and treatment is guided by a standardized patient pathway. Patients with minor stroke and TIA are generally hospitalised for observation for 72 h.

### Study and recruitment procedures

**Fig. 1:**
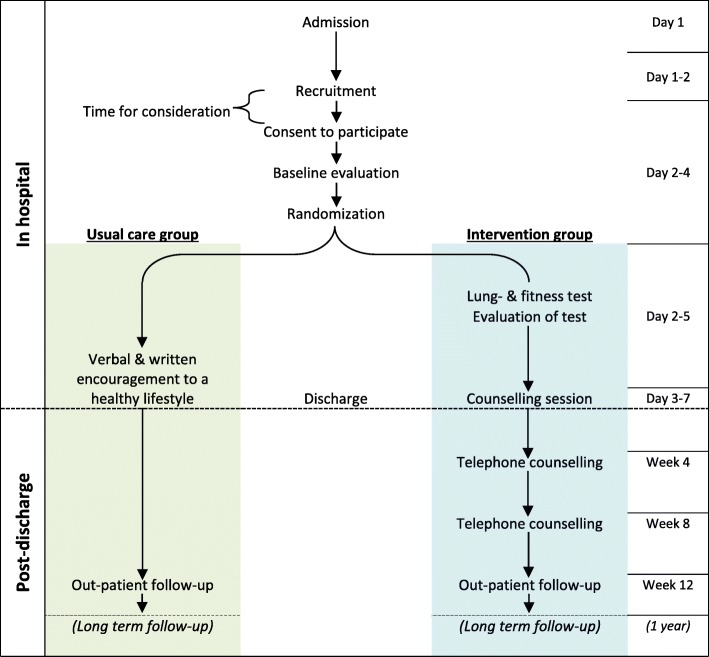
Flowchart of recruitment, inclusion, allocation and follow-up of participants

All new patients at the Department of Neurology will be screened for eligibility by the research investigator (JL). Patients are eligible if they are ≥ 18 years old, are diagnosed with TIA (ICD-10 G45.9) or minor stroke (ICD-10 I61, I63, I64; Scandinavian Stroke Scale 45-58), are discharged to their home, and can provide valid written consent. Diagnoses must be confirmed by a neurologist. Patients are excluded from the study if they have severe communication barriers, are not able to use a telephone, have severe disability prior to the stroke (WHO Performance Status > 2; incapable of self-care and mobilised less than 50% of the day), have an active abuse of alcohol or narcotics or have a severe psychiatric illness (affective disease, dementia, schizophrenia, anxiety). Eligible patients will be invited to participate through verbal and written information regarding the study purpose and method by JL. All participants must give written informed consent before participation. Participant flow is summarised in Fig. 1.

### Randomization and group allocation

Simple non-stratified 1:1 randomisation will be used to allocate participants into two trial arms (intervention or usual care) after baseline testing. Randomization will be conducted in the Research Electronic Data Capture (RedCap) software [19] using a computer generated randomization sequence. The randomization sequence will be generated by JL, then concealed in RedCap, and any changes can only be made by an external administrator. Participants will be automatically randomized after baseline testing and the allocation will be subsequently locked and cannot be altered. Participants will remain in the allocated arm for the entire intervention period.

#### Intervention

**Fig. 2:**
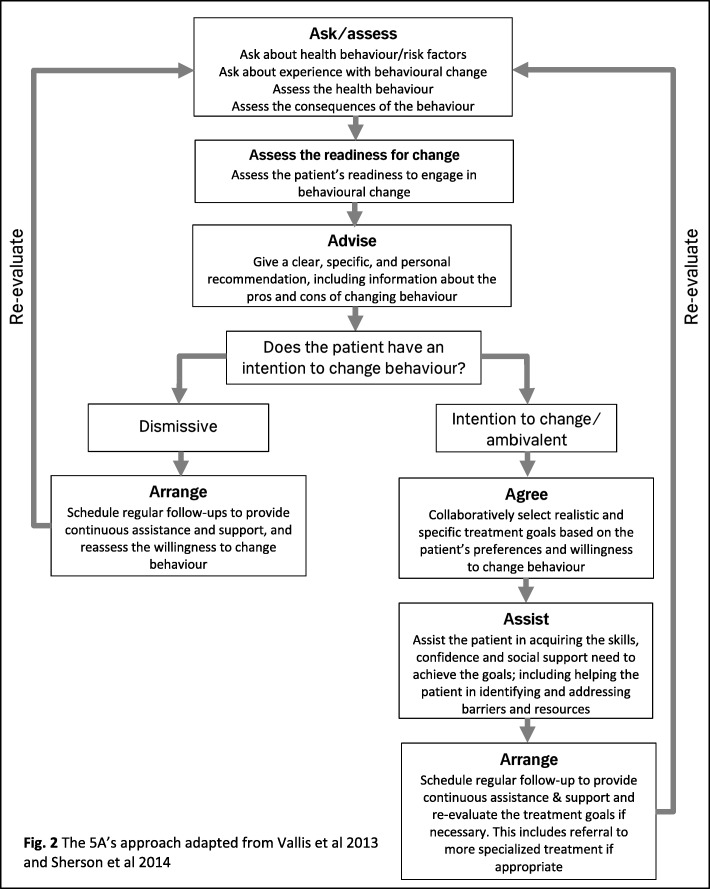
The 5A’s approach adapted from Vallis et al 2013 and Sherson et al 2014

Participants in the intervention arm will receive usual care and a nurse-led targeted lifestyle counselling focusing on smoking cessation, everyday physical activity, and adherence to preventive medication. The initial counselling will be provided face-to-face by a research investigator before the participant is discharged from the hospital. The aim of the counselling will be to engage the participant to partake a healthy lifestyle and adhere to the preventive medication and to assist the participant in finding suitable strategies to achieve his/her goals. Participants will be encouraged to bring a relative or close friend to the initial counselling session, who can be a support for the participant after discharge. The counselling will be based on the 5A’s approach (Ask/assess, Advice, Agree, Assist and Arrange, see Fig. 2) [20, 21]. *Ask/assess*. At baseline all participants will be asked about self-rated health, smoking habits, and physical activity, and body mass index, waist/hip-ratio and lung capacity will be assessed. During the counselling, the participants are asked to reflect on the following: the relevance of changing behaviour; how the specific behaviour affects their risk of disease; how they think changing behaviour could be beneficial as well as perceived barriers and facilitators of behavioural change. Perceived barriers and facilitators for changing behaviour can be both personal (e.g. attitudes towards changing behaviour, attribution of risk and self-efficacy) and environmental (e.g. products, technology, surroundings, social support and services). The focus at this point in the counselling is not to find solutions, but to facilitate the participant’s reflection on his or her health and health behaviour. *Advise*. The participant is given clear, specific and personal recommendations, including information on potential harms and benefits to their health and well-being. *Agree*. The participant is supported in setting goals. *Assist*. The investigator assists the participant in finding strategies to achieve the agreed-upon goals and helps the participant in acquiring the skills, confidence and social support within the participant’s own environment to change behaviour. The strategies should be grounded in the previous reflections and customised to the needs, capacities, and preferences of the individual participant. *Arrange*. Follow-up sessions are arranged and, if necessary, the participant is referred to other relevant treatments.

Prior to of the initial counselling session a detailed assessment of the participant’s lifestyle and physical condition will be conducted by JL (research investigator); including spirometry (FEV1/FVC measured using SpiroBank II) [22] and aerobic capacity (Åstrand-Rhyming Bike Test) [23].

Further counselling will be provided by telephone 4 and 8 weeks after discharge and will aim at maintaining motivation and adjusting goals and strategies if necessary. If the participant has not previously been motivated for changing behaviour, the willingness for change is re-assessed.

All parts of the intervention will be delivered by JL, who has extensive clinical experience as a nurse in the field of neurology.

After each counselling session, a written summary is included in the electronic patient record which is available to the participant via a personal log-in. The participant’s relatives will be involved in the counselling at the participant’s discretion to facilitate social support. Social support might be of particular importance in participants with cognitive impairments or limited ability to take in information.

The level of physical activity in the intervention arm will be monitored throughout the intervention period using an electronic activity tracker (Garmin VivoFit 4), which measures steps, intensity (light/moderate/vigorous activity) and estimated calories burned. The activity tracker should work as a reminder and motivator for everyday physical activity but will also provide feedback to support the post-discharge counselling.

#### Usual care

Participants in the control arm will receive standard care, which includes a review of prescribed medication, and both verbal and written encouragement of a healthy lifestyle (see Table 1) [24].

**Table 1:** Standard recommendations for a healthy lifestyle after stroke or TIA in Denmark [24]

• Smoking cessation is encouraged • Regular physical activity is encouraged to the extent of the patient’s ability • Patients with a substantial use of alcohol (> 7/14 units of alcohol per week for women and men, respectively) should reduce the consumption of alcohol or cease to use alcohol all together • Patients are encouraged to eat a diet high in fruits, vegetables, wholegrain products, and sea food; and to limit the intake of salt and saturated fats	

Participants from both treatment arms will be re-assessed in the hospital-based outpatient clinic 12 weeks after they are discharged from the hospital. The evaluation will include arterial blood pressure, smoking status, body mass index, waist/hip-ratio, and adherence to preventive medication (proportion of missed doses in the past 7 days) Fig 2.

### Study tests and assessments

Baseline and outcome measures are summarised in Table 2.

**Table 2:** Baseline and outcome parameters

Parameters	Methods	Baseline test	Discharge	12-weeks follow-up	1-year follow-up
**Baseline data**					
Demographic data		X			
Age, gender, living conditions, education, performance status [25] prior to stroke					
Health status					
Stroke type	ICD-10 I61, I63, I64, G45.9	X	X		
Stroke severity	Scandinavian Stroke Scale [26]	X			
Vital signs	Early warning score [27]	X			
Heart arrhythmia	Result of 48-72 h telemetry [28]		X		
Prior health problems	Charlson comorbidity index [29]	X			
Biochemistry	Glucose, HbA1c, cholesterol, LDL, VLDL, HDL, triglycerides [11]		X		
Prescribed medication	Prescribed preventive medication (Antihypertensives, antithrombotic, anticoagulatives, NOAC, statins)		X		
**Primary outcome**					
Eligibility rate		X			
Study participation rate		X			
Adherence to the program				X	
Attrition rate				X	
Satisfaction				X	
**Secondary outcome**				X	
Resting arterial blood pressure	Average of two or more measurements in sitting position after > 10 min resting according to 2017 US Guidelines [30]	X		X	
**Tertiary outcomes**					
Current smoking	Self-reported tobacco smoking (daily, weekly, rarely, has quit smoking, never smoked) [31]	X		X	
Physical activity	Self-reported participation in leisure time physical activities (minutes per week of light/moderate/strenuous) [32]	X		X	
Body composition	Body mass index, hip/waist ratio	X		X	
Fatigue	Fatigue Assessment Scale [33]; 10-item questionnaire	X		X	
Self-rated health	Self-rated health current and in the last year [31]	X		X	
Adherence to preventive medication	Adherence to preventive medication (Antihypertensives, antithrombotic, anticoagulatives, NOAC, statins) the last 7 days prior to assessment			X	
Units of alcohol per week	Self-reported [31]	X		X	
Recurrent stroke/TIA	Patient medical report data				X
Ischemic heart disease	Patient medical report data				X
All-cause mortality	Danish Central Person Registry				X

*LDL* low-density lipoprotein, *VLDL* very low-density lipoprotein, *HDL* high-density lipoprotein, *NOAC* novel oral anticoagulants

#### Baseline data

After written consent has been obtained baseline data will be collected by the research investigator. Demographic data will include age, gender, living arrangements, educational level and ECOG performance status [25] prior to stroke onset. Lifestyle factors will include self-rated health, smoking habits, alcohol consumption and anthropometric measures, assessed using standardised questions from the Danish National Health Survey Questionnaire [31]. The International Physical Activity Questionnaires short form (IPAQ-SF) [32] will be used to assess the level of physical activity prior to admission. Scandinavian Stroke Scale [26] will be used to assess stroke severity. Charlson Comorbidity Index [29] will be used to assess the burden of comorbidities. Early Warning Score [27] will be used to summarize vital signs. Serum lipids (total cholesterol, HDL, LDL, VLDL, triglycerides), random blood glucose and HbA1c are routinely collected on all patients [11].

### Outcome measures

#### Measures of feasibility

The primary outcome measures are the following:

1. the eligibility rate (proportion of eligible patients compared to the total number of stroke patients)
2. the study participation rate (proportion of patients accepting participation in the study)
3. the degree of adherence to the program (proportion of attendance in follow-up sessions)
4. attrition (drop-out and withdrawal)
5. participant satisfaction with the intervention, using both quantitative (Likert scale) and qualitative (semi-structured interviews) measures, to guide the design of a future full-scaled randomized controlled trial

#### Measure of potential effect

The secondary outcome measure will be *arterial blood pressure* measured before and 12 weeks after the start of the intervention. Measurements will be performed in accordance with the American Heart Association guidelines [30]. To avoid extreme values, the blood pressure will be measured at least twice. If the two measurements of the systolic pressure are more than 5 mmHg apart the measurement will be repeated. The average of the last two measurements will be used for analysis.

#### Other outcome measures

Tertiary outcomes after 12 weeks will include the following: self-reported smoking status, physical activity level (IPAQ-SF) [32], adherence to medication (number of missed/consumed doses in the past seven days), anthropometrics (body weight, waist/hip ratio) and Fatigue Assessment Scale [33].

#### Long term outcome

All participants will be followed up twelve months after the incident stroke/TIA through the national patient registry, which contains information on all patient admissions in Danish hospitals. The outcome measure will be recurrent strokes/TIA, myocardial infarcts, other cardiac admissions and death.

### Analysis plan

#### Quantitative data

Data will be entered into a RedCap database in real-time using electronic case report forms. Statistical analyses will be carried out using R 3.3.1/R Studio 0.99.

Recruitment, randomization, allocation and follow-up will be reported in a flow-chart stating the number of participants at each step. Characteristics of participants and non-participants (e.g. excluded, non-consenting or lost to follow-up) will be reported as detailed as possible according to CONSORT guidelines for feasibility studies [34].

Baseline data on participant demographics and characteristics will be presented in a descriptive table in accordance with the CONSORT guidelines.

The primary endpoint (12 weeks study retention in the two randomization groups) will be reported as absolute numbers and proportions with 95% confidence intervals. The secondary endpoint (arterial blood pressure) will be reported as an absolute number for each group and estimate of the between group difference with 95% confidence intervals. Twelve months follow-up data will be reported as absolute numbers, absolute risk reduction and risk ratio (with 95% confidence intervals) comparing the risks of a negative event (recurrent stoke, TIA, cardiovascular incident or death) in the two groups. An intention-to-treat approach will be employed in the analysis of all endpoints.

#### Qualitative data

Based on purposeful sampling [35] selected participants from both randomization groups will be invited to participate in in-depth semi-structured interviews. The participants are encouraged to invite a relative to participate in the interview. The sampling strategy will aim at achieving maximal variation concerning sex, time since discharge, and whether they have been successful in changing behaviour. The sample size will be based on achieving theoretical data saturation (expected *n* = 15 − 20). A semi-structured interview guide will be designed to give structure to the interviews.

The interviews will be digitally recorded and transcribed, and analysed using thematic analysis as described by Braun and Clarke [36]. The qualitative software system NVivo version 12 (QSR International) will be used to organize data and support the process of analysis.

### Approvals and registrations

The study protocol has been approved by the Scientific Committee of the Capital Region (H-17040484) and the Danish Data Protection Agency (j.nr. VD-2018-306, I-6552). The study protocol is registered at ClinicalTrials.gov (NCT03648957).

## Discussion

Most patients with TIA and minor stroke are discharged to their own home with little or no specialised follow-up. Previous studies have reported a high prevalence of cognitive impairments such as poor memory, fatigue and difficulty with reading and writing [3] as well as a high risk of stroke recurrence [37, 38]. The overall purpose of the current study is to develop an early initiated, client-centred behavioural intervention to support patients with minor stroke and TIA in managing their own health post-discharge. The intervention is pragmatically integrated within the existing standard treatment and guidelines.

Self-management of health is complex and requires both knowledge, skills, and confidence [39]. Optimizing counselling and providing formal support from health professionals might help patients obtain a greater adherence to lifestyle recommendations and medication prescriptions.

The rationale behind early initiation of the intervention was an assumption that patients might be more susceptible to information and behavioural change close to the initial event. In studies of other patient populations, it has been hypothesized that the time close to the initial diagnosis constitutes a certain *window of opportunity*, i.e. a limited period of time where the patient is more receptive to information [18]. In previous studies of behavioural interventions in patients with stroke and TIA, it has been shown that the time of inclusion has varied from a few days to several years post-stroke [15–17]. This might be part of the reason why results have been inconsistent.

Part of the purpose of the pilot study will therefore be to examine whether the patients are willing and able to participate in this type of intervention in the early phase of their disease. We anticipate that some patients will not be ready to participate at this stage, either because they find it too overwhelming to participate or because they are unable to take in the amount of information needed to engage in behavioural change.

Furthermore, we will examine whether it is feasible to initiate and complete the counselling within the limited time of admission in an acute setting. Patients with minor stroke and TIA are generally hospitalized for 3-4 days (72 h of observation) and this time frame might prove to be difficult to identify and include relevant patients in the study and provide the intervention.

Smoking, physical activity, and adherence to preventive medication were chosen as the main focus of the counselling intervention as these factors have been shown to be both modifiable [40, 41] and, in our judgement, were the most likely to have an impact on the systolic blood pressure [42, 43].

Systolic blood pressure was chosen as the main measure of effect because it is the greatest risk factor of both ischemic and haemorrhagic stroke [7] and has been demonstrated to be modifiable in previous intervention studies [12].

The randomized controlled design will be used to test the feasibility and acceptability of the overall study methodology and the specific elements of the intervention. This includes testing whether the screening procedure is suitable for identifying relevant patients, inclusion and exclusion criteria has the right balance of sensitivity and specificity, the procedure of recruiting and attaining consent is suitable, and delivery of the intervention and follow-up is acceptable. Through this process, we will gain knowledge and experience which can guide further development of the study protocol [44].

The qualitative interviews with the study participants will be used to gain insights into the lived experience of life going home after hospitalisation for stroke, as well as their experience of managing their own health. This will give us a more profound understanding of the needs of patients who are discharged from the hospital and which type of support and counselling should be provided before and after the discharge.

Furthermore, the qualitative interviews will be used to evaluate the randomized controlled trial and the intervention. Even though this area of research is relatively new, previous studies have found that engaging patients and other stakeholders in the development of clinical studies might help the researcher in developing more meaningful and feasible methods and to identify more useful outcome measures [45]. The qualitative interviews might also contribute with a deeper and more nuanced understanding of the barriers and facilitators of health enhancing behaviour in patients with stroke. The needs of this particular group of patients might differ from other patient groups because of the combination of older age and cognitive impairments.

A key part of a pilot study is to evaluate the feasibility of conducting a full-sized trial in the future.

A potential impediment for conducting a full-sized trial of this type might be a restricted number of willing participants. To assess whether a full-sized trial is feasible, we must therefore (1) assess how many participants can realistically be recruited within a reasonable time period, and (2) analyse available data on non-participants and low adherence to find potential strategies to reduce non-participation.

### Limitations

The present study will have some limitations. First off, we wish to evaluate the feasibility of the study design and the intervention, and the sample size of the study will therefore be limited increasing the risk of both type 1 and type 2 error. The sample size of 40 participants was not based on a formal sample size calculation, but rather on a pragmatic estimate of how many participants we would be possible to recruit within a reasonable timeframe, knowing full well that the trial would likely be under-powered for hypothesis testing. Though we assumed that 40 participants would be enough to reliable test all procedures of the trial even with a substantial rate of attrition.

The measurement of blood pressure will be done according to a standardized study manual to increase the validity and reliability. Self-reported data at baseline and follow-up will be collected using the same method in both treatment groups to minimize information bias.

Given the nature of the intervention the possibility of blinding is limited. Blinding of the assessors or participants is not possible in studies in which patient activation and counselling is part of the intervention. However, blinding in the analysis of the quantitative data will be possible.

### Ethical considerations

The study will be conducted in accordance with the Helsinki Declaration [46] including respect for the participants’ autonomy and right to informed consent. Participants will be informed that participation is voluntary, and that participation can be declined at any time and without explanation. Participant data will be kept confidential in accordance with guidelines from the Danish Data Protection Agency.

The inconveniences of participating will be minor, and we are convinced that the potential benefits will outweigh the drawbacks. Withdrawal symptoms associated with smoking cessation and muscle soreness associated with increased physical activity are to be expected. Generally, these will be both minor and transient [47, 48]. Participants will be encouraged to report any suspected adverse effects, including side effects to the medication as this might affect adherence. At all follow-up contacts (telephone and outpatient), the participants will be asked if they have experienced adverse effects or side effects to the medication. If potentially serious adverse effects are reported, a department physician will be consulted. All reports of adverse or side effects will be qualitatively evaluated and quantitatively compared between the trial arms.

The use of a control group as a comparison is necessary if we wish to find evidence of the potential hypothesized effect of the treatment and gain a better understanding of the variation in the outcome measures. It will also be necessary to test the feasibility of the randomization process prior to the design of a full scale randomized controlled trial. The treatment of the participants in the control group will at no point be inferior to the treatment of non-participants.

### Dissemination

Results of the study will be published in international peer-reviewed scientific journals and presented at national and international conferences. Results will be made public regardless of them being positive, negative, or inconclusive.

The principal investigator (JL) will draft all publications under the supervision of the other members of the project group. The order of authorship will be in order of contribution and has been agreed upon in the project group beforehand. The rights and responsibilities of the authors will be in accordance with *The Danish Code of Conduct for Research Integrity* [49] and the ICMJE recommendations [50].

### Trial status

Recruitment has been completed in January 2020.

## Data Availability

Data used during the current study will be available from the corresponding author upon reasonable request.
